# 2D ultrasonography and contrast enhanced ultrasound for the evaluation of cavitating mesenteric lymph node syndrome in a patient with refractory celiac disease and enteropathy T cell lymphoma

**DOI:** 10.1186/1471-230X-13-26

**Published:** 2013-02-11

**Authors:** Cristina Pojoga, Lidia Ciobanu, Alexandru Florin Badea, Emil Boţan, Cosmin Caraiani, Claudia Hagiu, Grigore Băciuţ, Radu Badea

**Affiliations:** 1 “Octavian Fodor” Regional Institute of Gastroenterology and Hepatology, 19-21, Croitorilor Street, 400 162, Cluj-Napoca, Romania; 2 “Iuliu Hatieganu” University of Medicine and Pharmacy, 8, Victor Babes Street, 400 012, Cluj-Napoca, Romania

**Keywords:** CEUS, Celiac disease, Peripheral T-cell lymphoma, Cavitating mesenteric lymph node syndrome

## Abstract

**Background:**

The cavitating mesenteric lymph node syndrome (CMLNS) is a rare manifestation of celiac disease, with an estimated mortality rate of 50%. Specific infections and malignant lymphoma may complicate its clinical course and contribute to its poor prognosis. Diagnosing the underlying cause of CMLNS can be challenging. This is the first report on contrast enhanced ultrasound (CEUS) findings in enteropathy associated T-cell lymphoma (EATL) complicating CMLNS in a gluten-free compliant patient with persistent symptoms and poor outcome.

**Case presentation:**

We present the case of a 51-year old Caucasian male patient, diagnosed with celiac disease and CMLNS. Despite his compliance to the gluten-free diet the symptoms persisted and we eventually considered the possible development of malignancy. No mucosal changes suggestive of lymphoma were identified with capsule endoscopy. Low attenuation mesenteric lymphadenopathy, without enlarged small bowel segments were seen on computed tomography. CEUS revealed arterial rim enhancement around the necrotic mesenteric lymph nodes, without venous wash-out. No malignant cells were identified on laparoscopic mesenteric lymph nodes biopsies. The patient died due to fulminant liver failure 14 months later; the histopathological examination revealed CD3/CD30-positive atypical T-cell lymphocytes in the liver, mesenteric tissue, spleen, gastric wall, kidney, lung and bone marrow samples; no malignant cells were present in the small bowel samples.

**Conclusions:**

CEUS findings in EATL complicating CMLNS include arterial rim enhancement of the mesenteric tissue around the cavitating lymph nodes, without venous wash-out. This vascular pattern is not suggestive for neoangiogenesis, as arteriovenous shunts from malignant tissues are responsible for rapid venous wash-out of the contrast agent. CEUS failed to provide a diagnosis in this case.

## Background

Cavitating mesenteric lymph node syndrome (CMLNS) is a rare, poorly understood complication of celiac disease characterized by central necrosis of mesenteric lymph nodes [1]. Multiple areas of necrotizing cavitation in reticulo-endothelial tissues are frequently associated with splenic hypo-function, the presence of Howell-Jolly bodies, monocytosis, lymphocytosis and increased platelet count [1,2]. The estimated mortality rate is 50% [1]. Enteropathy associated T cell lymphoma (EATL) is a possible complication of the necrosis of mesenteric lymph nodes [2,3]. Hepatic failure is a rare complication of celiac disease of unknown pathogenesis; the predisposition to autoimmunity may, in part, explain the involvement of the liver [4]. In patients associating CMLNS, septicemia or malignant T-lymphocyte infiltration of the liver may explain the liver insufficiency [2,3].

Diagnosing the underlying condition of the cavitating mesenteric lymph node syndrome (either infectious or malignant) can be challenging; a lymphoma can still be notoriously difficult to diagnose, despite multiple biopsies [5]. Even lymphomas with T-cell immunophenotypic features have been reported in extra intestinal sites (liver, spleen), complicating celiac disease, but without intestinal involvement [2].

Computed tomography (CT) or magnetic resonance imaging (MRI) can detect fat-fluid levels in mesenteric cystic masses, considered as specific findings for CMLNS [6,7]; furthermore, these techniques can identify a segmental enlargement of the small bowel, suggestive for lymphoma. More recently developed, contrast enhanced ultrasound (CEUS) can depict the blood flow in small vessels, under 40 microns, useful to describe the tumor angiogenesis patterning [8]. The intense, anarchic arterial enhancement with rapid venous wash-out of the contrast agent is highly suggestive for malignancy, since the malignant tissue is characterized by numerous arteriovenous shunts [8].

We report a case of celiac disease complicated with CMLNS, with persisting symptoms despite adherence to the gluten-free diet. The imaging and histological examinations failed to document the presence of malignancy during the patient’s lifetime. A fulminant liver failure caused the patient’s death 14 months after the initial diagnosis. The postmortem histopathological examination revealed abnormal T-cell lymphocytes in the liver, mesenteric tissue, spleen, gastric walls, kidney, lungs and bone marrow; no malignant cells were present in the examined small bowel samples.

## Case presentation

**Figure 1:**
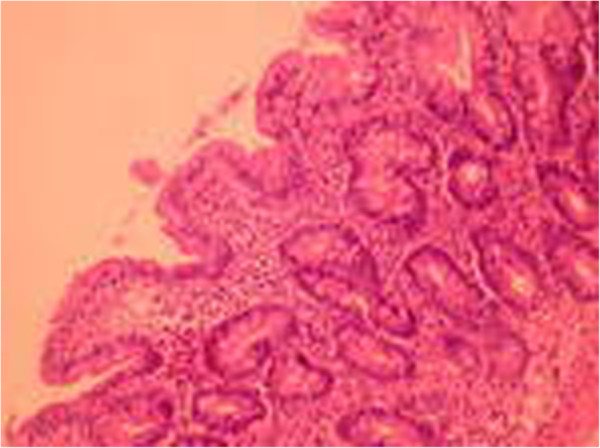
Duodenal biopsy: villous atrophy, crypt hyperplasia, intraepithelial lymphocytes without atypical features.

**Figure 2:**
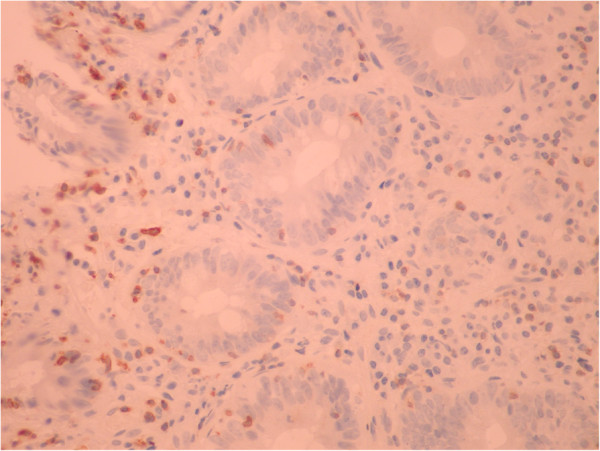
Immunohistochemistry testing from initial duodenal biopsy: intraepithelial lymphocytes positive for CD3.

**Figure 3:**
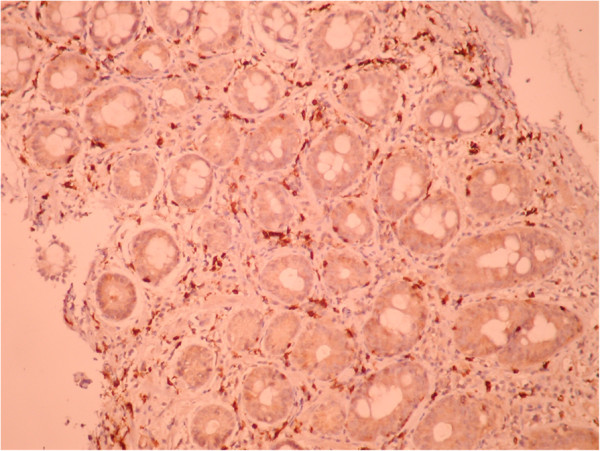
Immunohistochemistry testing from initial duodenal biopsy: few lymphocytes positive for CD8 in lamina propria.

**Figure 4:**
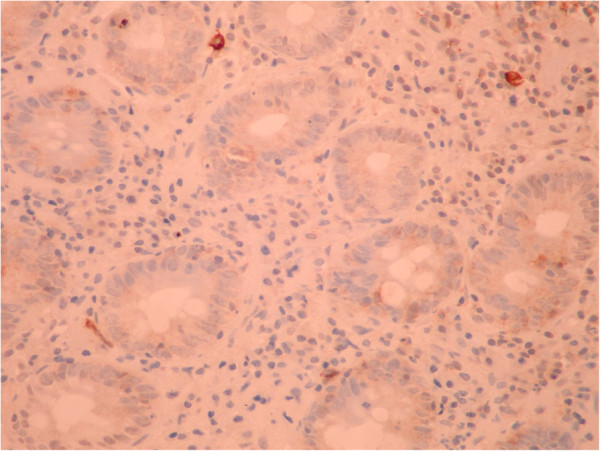
Immunohistochemistry testing from initial duodenal biopsy: negative CD30 staining.

**Figure 5:**
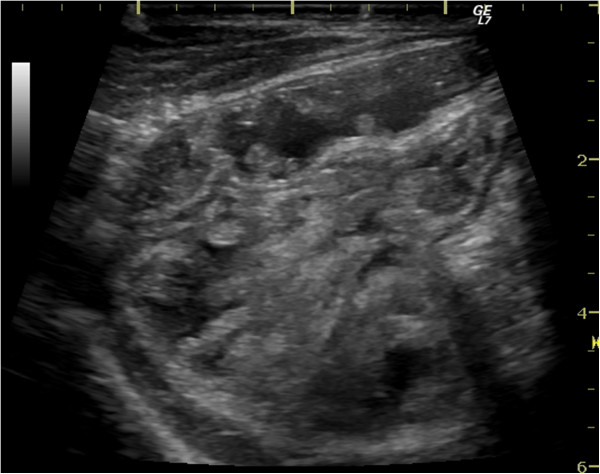
Abdominal ultrasound – intestinal loops with enlarged, hyperechoic mesentery.

A 51-year old male was investigated for chronic diarrhea, episodes of moderate diffuse abdominal pain and 10-kg weight loss. On physical examination, the patient presented muscle wasting, without any fever, hepatosplenomegaly or jaundice. The stool studies were positive for steatorrhea. The laboratory workup revealed moderate iron deficiency anemia, signs of hyposplenism: Howell-Jolly bodies on the peripheral blood smear, elevated platelet count, hypocalcaemia and an elevated alkaline phosphatase level. The serum endomysial and tissue transglutaminase IgA antibodies were positive in high titre. Total villous atrophy, crypt hyperplasia, increased intraepithelial lymphocytes and increased plasma cells and lymphocytes in the lamina propria were found on the duodenal biopsy (Figure 1) performed by upper digestive endoscopy. The intraepithelial lymphocytes were small, without atypical features. The immunohistochemistry testing found intraepithelial lymphocytes positive for CD3 (Figure 2) and few lymphocytes positive for CD8 in lamina propria (Figure 3); CD30 staining revealed isolated positive cells in lamina propria (Figure 4). The abdominal ultrasound revealed fluid-distended small bowel loops with an enlarged, hyper echoic mesentery (Figure 5), anechoic cysts corresponding to mesenteric lymph nodes and a reduced spleen size. We established a diagnosis of celiac disease complicated with CMLNS. A gluten-free diet was recommended and a three-month monitoring schedule proposed.

Three months later, the patient complained of persistent symptoms. The stool examination revealed a Yersinia enterocolitica infection. The patient received adequate antibiotic therapy, resulting in stool sterilization.

**Figure 6:**
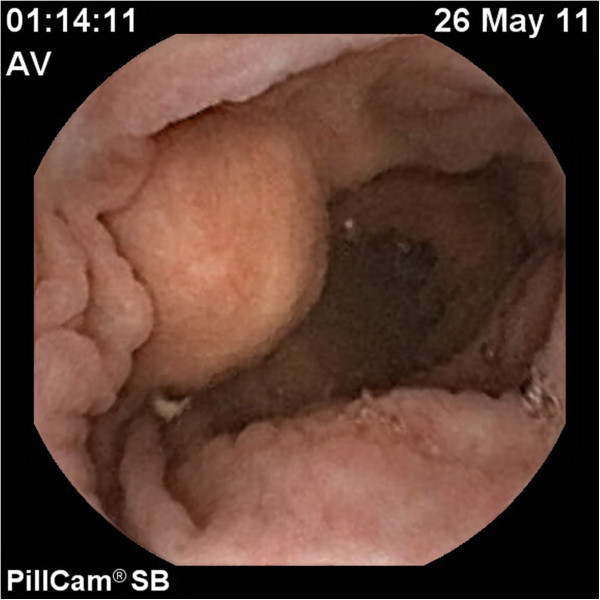
Small bowel capsule endoscopy – “bulging mass” without superficial erosions of the mucosa – (compressive necrotic mesenteric lymph node).

**Figure 7:**
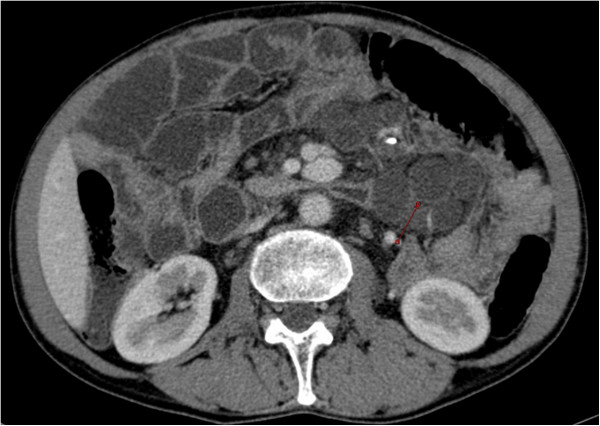
Computed tomography – cystic mesenteric masses - central low attenuation with enhanced rims (arrow).

**Figure 8:**
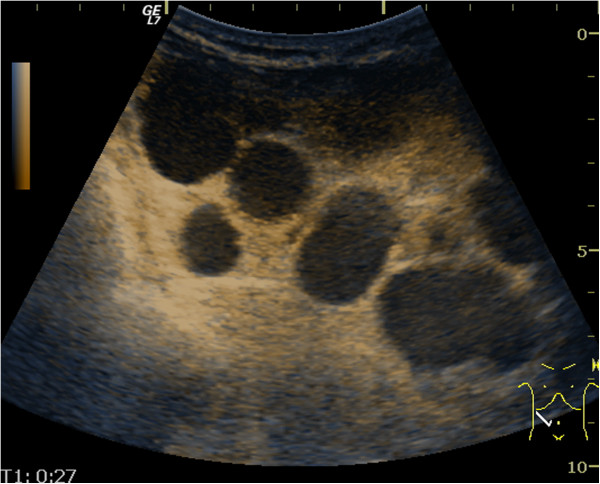
Contrast-enhanced ultrasound - arterial phase (27 seconds) – cystic mesenteric masses - arterial rim enhancement.

**Figure 9:**
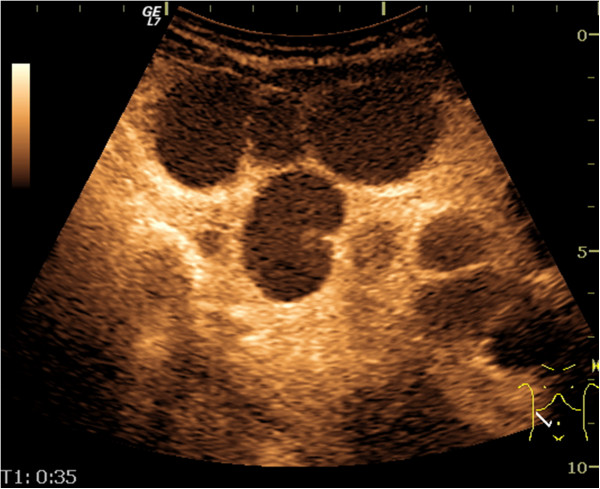
Contrast-enhanced ultrasound - venous phase (35 seconds) – cystic mesenteric masses - no was/out of contrast agent.

After six months of gluten-free diet, the clinical manifestations were similar, despite diet adherence, confirmed by the decline in tissue transglutaminase IgA titre. An intestinal lymphoma was suspected and capsule endoscopy performed, which investigated the entire small bowel. This examination revealed an atrophic villous pattern in the proximal jejunum, without mucosal changes suggestive of lymphoma; a “bulging” mass with normal mucosal surface was described (Figure 6) and was interpreted as compression from a mesenteric lymph node. The entire small bowel was investigated by capsule endoscopy. Mesenteric cystic masses with central low attenuation and a thin enhancing rim were found on oral and IV-contrast enhanced computed tomography (CT) (Figure 7). No small bowel wall segmental enlargements were present on CT enteroclysis. As the clinical suspicion of malignancy was still high, a contrast-enhanced ultrasound (CEUS) was considered in order to describe the vascular pattern of the mesenteric tissue. After peripheral venous injection of 4.8 ml of ultrasound contrast agent (Sonovue), an arterial rim enhancement (Figure 8) was seen around necrotic lymph nodes, without washout of the contrast agent in the venous phase (Figure 9). Some of the investigated masses had septa exhibiting the same vascular pattern, suggesting an anarchic vascularization. A diagnostic laparoscopy was performed with removal of two lymph nodes. These cystic masses were found to contain a milky fluid. The histopathological examination of the samples revealed central homogeneous acidophilic material, fibrotic walls with a rim of normal lymphocytes at the periphery of necrotic lymph nodes and no signs of malignancy or infection. The gluten-free diet and monitoring was continued.

After another five months, the patient presented with fever (39°Celsius) and severe liver failure. The blood cultures were negative. The serological markers for viral or autoimmune hepatitis and leptospirosis were also negative. The already severe clinical status worsened, with the development of hepatic encephalopathy and severe upper gastrointestinal bleeding. Despite intensive supportive treatment, the patient died 48 hours after admission.

**Figure 10:**
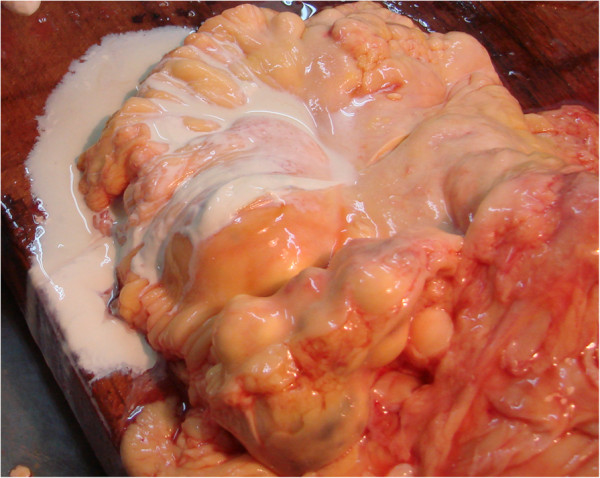
Necropsy gross findings - many nodular grayish-white masses in the mesentery; a milky liquid was observed after section.

**Figure 11:**
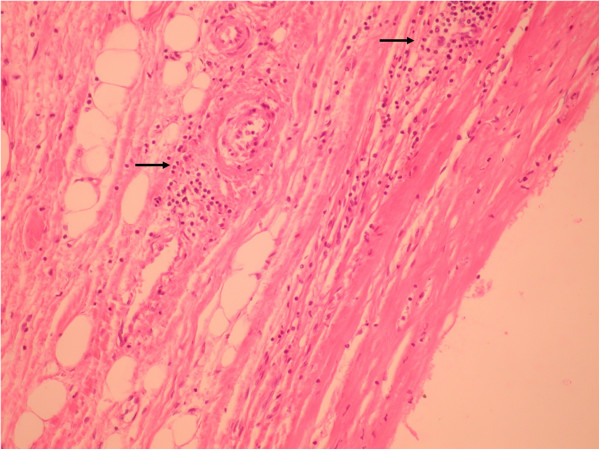
Histological examination (Hematoxylin-Eosine staining, 20x) mesenteric pseudo-cyst – central homogeneous acidophilic material, fibrotic walls, rare lymphocytes and plasmocytes in the periphery (arrows).

**Figure 12:**
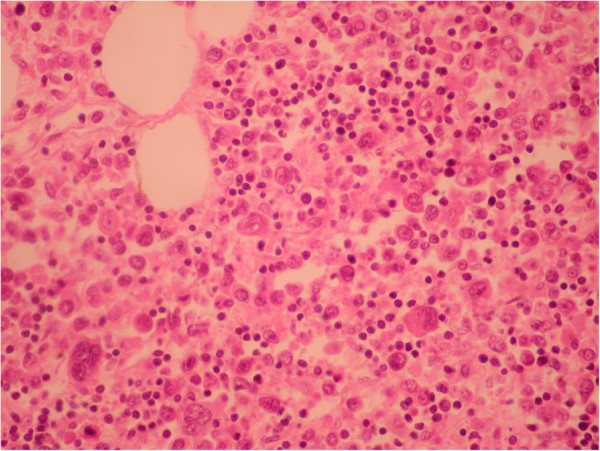
Mesenteric adipose tissue - Hematoxylin-Eosine staining, 20x: malignant lymphocytes.

**Figure 13:**
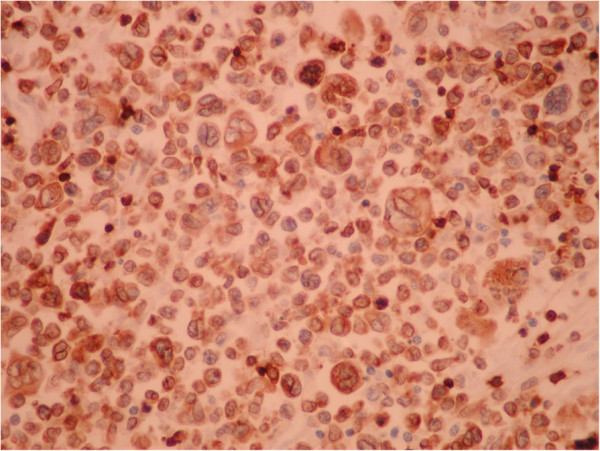
Mesenteric adipose tissue - Immunohistochemistry testing: mesenteric adipose tissue positive for CD3 (40x).

On necropsy, many nodular grayish-white masses, either fluctuant or firm, containing a milky fluid, were found in the mesentery (Figure 10). The microscopic examination of necrotic mesenteric lymph nodes revealed a central homogeneous acidophilic material, fibrotic walls and rare lymphocytes and plasmocytes in the periphery (Figure 11). Infiltration of abnormal T-cell lymphocytes, with atypical nuclear features, was present in the surrounding adipose tissue (Figure 12). The immunohistochemistry testing was positive for CD3 (Figure 13) and CD30. The same infiltrative tumor cells were present in the liver, spleen, gastric walls, kidney, lungs and bone marrow; no malignant cells were present in the small bowel samples examined.

## Discussion

The pathogenesis of CMLNS is not fully understood at the moment. One of the proposed theories involved an excessive antigenic exposure of the immune system via a damaged intestinal mucosa, leading to depletion of cellular lymphoid elements in the mesenteric lymph nodes and spleen, causing a cystic change or ‘cavitation’ in some patients with celiac disease. An alternative hypothesis is the necrosis of mesenteric lymph nodes triggered by localized immune-mediated complement activation and intravascular coagulation [7]. There were several reports of lymph node necrosis associated with intestinal infection with Mycobacterium spp, Yersinia spp [1] or Tropheryma whippelii [9]. Our patient had an infection with Yersinia enterocolitica during the course of his illness. However, this infection cannot be regarded as the cause of CMLNS, because the symptoms persisted even after adequate antibiotic treatment leading to stool sterilization.

Half of the CMLNS patients have a poor prognosis, but some reports mentioned a good outcome if a strict gluten-free diet was observed [10,11]. In celiac disease, however, a malignant lymphoma may also be the cause of mesenteric lymph node necrosis, being in part responsible for the poor prognosis [1].

The diagnosed of CMLNS is based on imaging and typical histopathological examination. The necrotizing mesenteric lymph nodes are described as anechoic cysts on an abdominal ultrasound. Computed tomography studies reveal central low attenuation with enhancing rims [6]. Sometimes fat-fluid levels within the masses may be apparent on CT [6]; this feature is considered unique to the cavitated mesenteric adenopathies associated with celiac disease. The specific fat-fluid levels were also found in one CMLNS case on an MRI examination [7].

The diagnosis of complicated cavitating mesenteric lymph node syndrome (either infectious or malignant) can be challenging; a lymphoma is still notoriously difficult to diagnose, despite multiple biopsies [5]. Lymphomas with T-cell immunophenotypic features have been reported in extra intestinal sites (liver, spleen), as a complication of celiac disease, but without intestinal involvement [2]. In our case, the initial biopsies did not reveal abnormal T-cell lymphocytes, but the virtual lack of immunopositivity for CD8 intraepitelial lymphocytes might have been suggestive of a diagnosis of refractory celiac disease type II [12]. Also the presence of isolated large CD30+ lymphocytes in the lamina propria in early biopsies could have represented minimal infiltration by the patient’s lymphoma [13].

The capsule endoscopy and CT did not find any small bowel mucosal changes suggestive of enteral lymphoma and no other small bowel biopsies were taken. The contrast-enhanced ultrasound was performed in order to obtain more specific information about the vascular pattern of the lymph node “walls” and the surrounding mesenteric tissue.

The assessment of neovascularization by CEUS is based on its ability to depict the blood flow in small vessels. In malignant tumors a rapid and intense enhancement is seen in the arterial phase, with rapid wash-out of the contrast agent in the venous phase. This vascular pattern is explained by arteriovenous shunts. The timing of hypo enhancing on CEUS may be correlated with tumor cell differentiation; well-differentiated tumors wash out more slowly than poorly differentiated ones [14]. The quantitative CEUS parameters in assessing neoangiogenesis processes were documented on hepatocarcinoma, ovarian and breast malignant tumors [15-17]. In malignant lymph nodes, CEUS depicts vessels penetrating the node’s capsule away from the hilum; reactive adenopathies have a singular vascular pedicle at the hilum with regular branches towards the periphery. Based on these findings, CEUS can improve the results of Doppler ultrasound in the differential diagnosis of lymph nodes with a sensitivity, specificity and accuracy rate of up to 84%, 79% and 80% respectively [18]. These studies were performed on superficial lymph nodes. However, in several studies, lymphomas seem to have a benign vascular pattern [19,20]. To date there is not enough evidence for the accuracy of CEUS in assessing different types of lymphoma [21]. In our case, intense enhancement was observed in the arterial phase in the surrounding mesenteric tissue of necrotic lymph nodes, without rapid vascular washout in the venous phase, but it was not suggestive of malignancy. CEUS failed to provide a diagnosis of tumor neoangiogenesis in this case. This lack of venous wash-out may be due to arborizing venules found in some peripheral T-cell lymphomas [22].

In this case, 18 F-FDG PET scan could have detected early EATL; previous studies documented its role in patients with refractory celiac disease, being more sensitive than CT in detecting sites affected by lymphoma [23]. This investigation was not available in our institution.

In patients associating CMLNS, liver failure may be due to septicemia or to malignant T-lymphocyte infiltration of the liver [2,3]. In our case, the microscopic examination revealed abnormal T-cell lymphocytes in the liver, spleen, mesenteric tissue, gastric walls, kidney, lung and bone marrow. No malignant cells were observed in the small bowel samples examined. It is possible that the EATL was unsampled, despite multiple biopsies, as it was previous reported [5]. Other authors described the same features of a rare peripheral T-cell lymphoma, associated with celiac disease, characterized by a rearrangement of the gamma-delta T-cell receptor, responsible for the aggressiveness of this tumor [2,24]. T-cell receptor PCR or flow cytometry were not performed in our case.

## Conclusions

This is the first report of a CEUS examination in CMLNS complicated with EATL that revealed an arterial rim enhancement around necrotic lymph nodes without venous wash-out. As this vascular pattern is not suggestive for tumor neoangiogenesis, the investigation failed to provide a diagnosis on this case.

## Consent

The consent for publication was signed by the patient’s son.

## Abbreviations

CMLNS: Cavitating mesenteric lymph node syndrome; CEUS: Contrast enhanced ultrasonography; EATL: Enteropathy-associated T cell lymphoma; CT: Computer tomography; MRI: Magnetic resonance imaging.

## Competing interests

The authors declare that they have no competing interests.

## Authors’ contribution

CP was the attending physician, analyzed and interpreted the data. LC was a major contributor in writing this manuscript and also interpreted the capsule endoscopy findings EB performed the autopsy and histological examinations (including the immunohistochemistry). CC interpreted the CT examinations. CH performed the ultrasound examinations. AFB and GB analyzed and interpreted the clinical and imagistic studies. RB has a major contribution in analyzing and interpreting CEUS findings. All authors read and approved the manuscript.

## Pre-publication history

The pre-publication history for this paper can be accessed here:

http://www.biomedcentral.com/1471-230X/13/26/prepub
